# Impact of Delivery Method on Initiation and Continuation of Breastfeeding: A Prospective Cohort Study

**DOI:** 10.3390/children12080966

**Published:** 2025-07-23

**Authors:** İlke Özer Aslan, Mustafa Törehan Aslan, Nebibe Can, Özlem Sevinç Ergül, Nihal Çallıoğlu

**Affiliations:** 1 Department of Obstetrics and Gynecology, Faculty of Medicine, Tekirdağ Namık Kemal University, Tekirdağ 59030, Türkiye; ozlem.sevinc@saglik.gov.tr; 2 Department of Obstetrics and Gynecology, In Vitro Fertilization and Reproductive Health Center, Acıbadem Maslak Hospital, İstanbul 34457, Türkiye; 3 Department of Pediatrics, Division of Neonatology, Faculty of Medicine, Tekirdağ Namık Kemal University, Tekirdağ 59030, Türkiye; torehanaslan@yahoo.com; 4 Department of Pediatrics, Division of Neonatology, Koç University Hospital, Istanbul 34010, Türkiye; 5 Maternity Ward, Faculty of Medicine Hospital, Tekirdağ Namık Kemal University, Tekirdağ 59030, Türkiye; necan@nku.edu.tr; 6 Department of Obstetrics and Gynecology, Division of Perinatology, Hamidiye Faculty of Health Sciences, Gaziosmanpaşa Training and Research Hospital, Istanbul 34255, Türkiye; niyalcll@hotmail.com

**Keywords:** breastfeeding continuation, breastfeeding initiation, cesarean section, emergency cesarean, planned cesarean, vaginal delivery

## Abstract

**Background/Objectives**: Cesarean delivery often leads to delayed breastfeeding initiation, potentially affecting infant health compared with vaginal delivery. This prospective observational study (conducted between August 2022 and January 2024) comparatively evaluates the impact of delivery method—vaginal, planned cesarean, and emergency cesarean—on breastfeeding initiation and continuation and examines the maternal factors influencing these outcomes. **Materials and Methods**: We enrolled 338 mother–infant pairs at a tertiary university hospital. Breastfeeding effectiveness was assessed using the Bristol Breastfeeding Assessment Tool (BBAT) at birth and at one, three, and six months postpartum. Rates of breastfeeding continuation and formula supplementation were documented through structured interviews. **Results**: The mothers who delivered vaginally had a significantly higher rate of breastfeeding within one hour after birth (85.5%) compared with planned (57.9%) and emergency cesarean sections (64.9%) (*p* < 0.001). Baseline BBAT scores were higher for vaginal births but converged across the groups by one month postpartum (*p* > 0.05). At six months, breastfeeding continuation rates remained high (94.4–95.2%) irrespective of delivery method. **Conclusions**: Delivery method exerts a transient effect on breastfeeding initiation. With lactation support, the mothers delivering by cesarean section achieved comparable breastfeeding outcomes within the first month postpartum. These findings reinforce the importance of Baby-Friendly Hospital Initiative (BFHI) practices, including immediate skin-to-skin contact, effective pain management, and lactation counseling, in ensuring equitable breastfeeding outcomes.

## 1. Introduction

Breastfeeding is universally recognized as a cornerstone of maternal and child health, providing optimal nutrition, enhancing immunity, and supporting cognitive and physical development in infants. It also confers significant maternal benefits, including a reduced risk of postpartum hemorrhage, certain cancers, and metabolic disorders [1,2]. Consequently, global health authorities such as the WHO and UNICEF recommend exclusive breastfeeding for the first six months and continuation up to two years or beyond, aligning with Sustainable Development Goals (SDGs) targeting child survival and well-being [3,4]. However, global rates of exclusive breastfeeding remain below the 60% target set for 2030, reflecting ongoing challenges in achieving equitable breastfeeding practices worldwide [5]. Delivery method has emerged as a key determinant of breastfeeding outcomes. Vaginal delivery facilitates immediate mother–infant bonding through natural hormonal surges, such as oxytocin release, which enhances lactation [6]. In contrast, cesarean section (C/S)—whether planned (elective) or performed emergently—can interfere with these physiological processes. Planned C/S, often scheduled before the onset of labor, lacks the hormonal priming of vaginal delivery, whereas emergency C/S, performed after labor begins, may partially preserve these effects but is frequently complicated by maternal fatigue, anesthesia, and surgical stress [7,8].

Current evidence suggests that both types of cesarean delivery are associated with the delayed initiation of breastfeeding compared with vaginal delivery, yet their relative impacts remain insufficiently explored [9,10]. Moreover, “Baby-Friendly Hospital” practices, including early skin-to-skin contact and immediate breastfeeding support, have been shown to mitigate some of the breastfeeding challenges associated with cesarean delivery [11,12]. Despite these interventions, cesarean delivery rates continue to rise globally, driven by both medical indications and non-medical factors, raising concerns about the potential downstream effects on breastfeeding practices [13]. Existing studies often fail to distinguish between planned and emergency cesarean deliveries when evaluating breastfeeding outcomes, leading to conflicting conclusions about the long-term impact of delivery method on breastfeeding continuation [14,15]. Furthermore, sociodemographic factors, such as maternal education and support systems, may modify these effects but are rarely controlled for in analyses [16,17,18].

This prospective evaluation of breastfeeding outcomes across delivery modes addresses a critical global health challenge, aligning with the WHO’s Global Breastfeeding Collective targets and the Sustainable Development Goals (SDG 2, 3, and 5) [6]. By distinguishing between planned and emergency cesarean deliveries, incorporating structured breastfeeding assessments, and controlling for key maternal and perinatal factors [19], this study provides nuanced insights into whether cesarean-related breastfeeding challenges are transient and modifiable. These findings have both methodological and practical significance for guiding targeted postpartum support interventions in settings with rising cesarean rates.

## 2. Materials and Methods

### 2.1. Study Design and Setting

This prospective observational cohort study was conducted between August 2022 and January 2024 at a tertiary Baby-Friendly hospital in Tekirdağ, Türkiye. The hospital’s designation as Baby-Friendly aligns with national policies supporting breastfeeding practices and the WHO’s Global Strategy for Infant and Young Child Feeding.

### 2.2. Ethical Considerations

This study was approved by the Tekirdağ Namık Kemal University Faculty of Medicine Ethics Committee (Approval No. 2022.135.07.02, Date: 26 July 2022). All procedures were conducted in accordance with the Declaration of Helsinki. Written informed consent was obtained from all participants. For mothers aged 16–17 years, consent from a legal guardian was additionally secured to ensure voluntary participation.

### 2.3. Participants

Participant inclusion criteria: Mothers aged 16–51 years who gave birth at ≥37 weeks of gestation during the study period and were willing to participate.

Exclusion criteria: Mothers with prior breastfeeding difficulties, previous breast surgery, or medical conditions contraindicating breastfeeding (e.g., HIV infection). The relatively high proportion of planned cesarean sections (52.7%) reflects local obstetric practices where elective C/S is often performed due to maternal request or perceived fetal risks, in line with national trends showing rising elective C/S rates.

### 2.4. Sample Size Calculation

Sample size calculation: The required sample size was calculated using G*Power 3.1 (Heinrich Heine University, Düsseldorf, Germany), targeting an effect size of 0.25, power of 80%, and alpha of 0.05 for detecting differences in breastfeeding outcomes across three delivery methods. A minimum of 300 mother–infant pairs was determined to be sufficient; 338 were ultimately enrolled.

### 2.5. Data Collection and Instruments

Breastfeeding effectiveness was assessed using the Bristol Breastfeeding Assessment Tool (BBAT), developed in 2015 [20]. The BBAT has been validated for use in Turkey [21] with a Cronbach’s alpha of 0.89 in our study population, indicating high internal consistency. The BBAT evaluates four domains, positioning, attachment, sucking, and swallowing, each scored from 0 (poor) to 2 (good), for a total score range of 0–8. A structured interview form, developed by the research team based on Ministry of Health guidelines, was used to collect sociodemographic data, breastfeeding continuation rates, and formula supplementation information.

### 2.6. Statistical Analysis

Data were analyzed using SPSS 27.0 (IBM Corp., Armonk, NY, USA). Descriptive statistics included means ± standard deviation (SD) and percentages. Normality was assessed via the Shapiro–Wilk test. One-way ANOVA with Bonferroni’s correction was used for continuous variables; chi-square tests for categorical variables. Repeated measures ANOVA evaluated changes in BBAT scores over time. Statistical significance was set at *p* < 0.05.

## 3. Results

A total of 338 mothers aged 16 to 51 years (mean 28.2 ± 4.2 years, range: 16–51) participated in the study. The majority were housewives (77.5%), and 17.8% had a university-level education. The mean pregestational BMI was 26.2 ± 3.2 kg/m^2^ (range: 19.1–34.8), and mean weight gain during pregnancy was 14.8 ± 4.8 kg (range: 7–27). The sociodemographic characteristics by delivery method are presented in Table 1.

**Figure 1:**
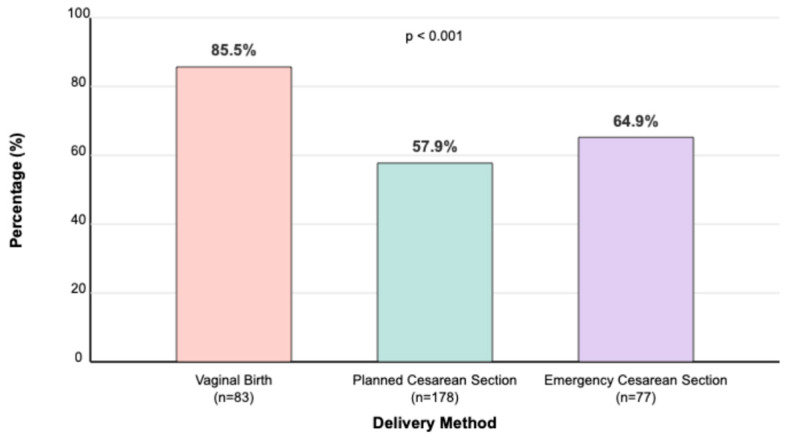
Proportion of mothers initiating breastfeeding within 1 h by delivery method. Percentage (%) of mothers initiating breastfeeding within 1 h. Delivery methods: vaginal birth, planned cesarean section, and emergency cesarean section. World Health Organization (WHO) [6] defines early initiation of breastfeeding as breastfeeding within the first hour after birth.

Overall, 98.5% of mothers initiated breastfeeding postpartum. However, the timing of initiation varied significantly by delivery method (*p* < 0.001): 85.5% of vaginal deliveries initiated breastfeeding within one hour, compared with 57.9% in planned cesarean sections and 64.9% in emergency cesarean sections (Figure 1). Over time, breastfeeding continuation rates remained high across the groups, with 95.9% at three months and 94.7% at six months postpartum. Concurrently, the use of formula supplementation and complementary foods gradually increased, reaching 17.8% and 24.3%, respectively, at six months. The proportion of mothers returning to work was minimal (0.6%) during the first three months, rising slightly to 4.4% by six months postpartum.

**Figure 2:**
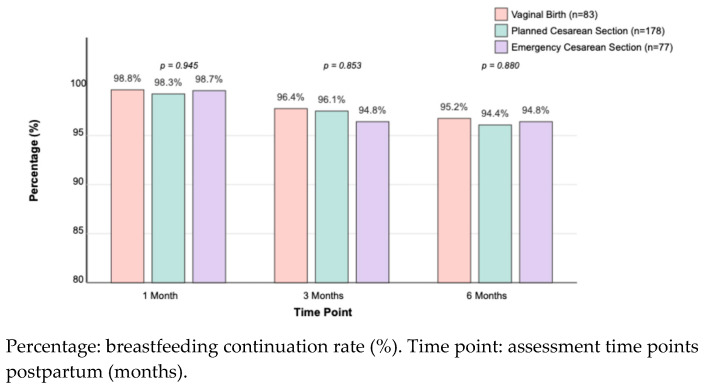
Breastfeeding continuation rates at 1, 3, and 6 months postpartum, by delivery method. Values represent the percentage of mothers continuing breastfeeding at each time point.

By one month postpartum, breastfeeding continuation rates were similarly high across the groups (vaginal: 98.8%, planned C/S: 98.3%, emergency C/S: 98.7%; *p* = 0.945). These high rates persisted at three months (vaginal: 96.4%, planned C/S: 96.1%, emergency C/S: 94.8%; *p* = 0.853) and six months postpartum (vaginal: 95.2%, planned C/S: 94.4%, emergency C/S: 94.8%; *p* = 0.880) (Figure 2). No significant differences were found between the groups in breastfeeding continuation rates at these later time points, highlighting that early disparities in initiation were transient (Figure 2).

At one month postpartum, formula supplementation rates were 13.3% in the vaginal delivery group, 16.9% in the planned C/S group, and 16.9% in the emergency C/S group (*p* = 0.724). These rates slightly increased by six months postpartum (vaginal: 16.9%, planned C/S: 18.0%, emergency C/S: 18.2%; *p* = 0.967). The proportion of infants receiving complementary foods at six months was also comparable across the groups (vaginal: 24.1%, planned C/S: 24.2%, emergency C/S: 24.7%; *p* = 0.995) (Table 2).

Significant differences were observed in baseline BBAT scores among the delivery methods. Vaginal deliveries had the highest mean total BBAT score at birth (1.56 ± 0.34; range: 0.9–2.0), followed by planned C/S (1.35 ± 0.33; range: 0.8–1.9), and emergency C/S (1.13 ± 0.38; range: 0.7–1.8) (*p* < 0.001).

**Figure 3:**
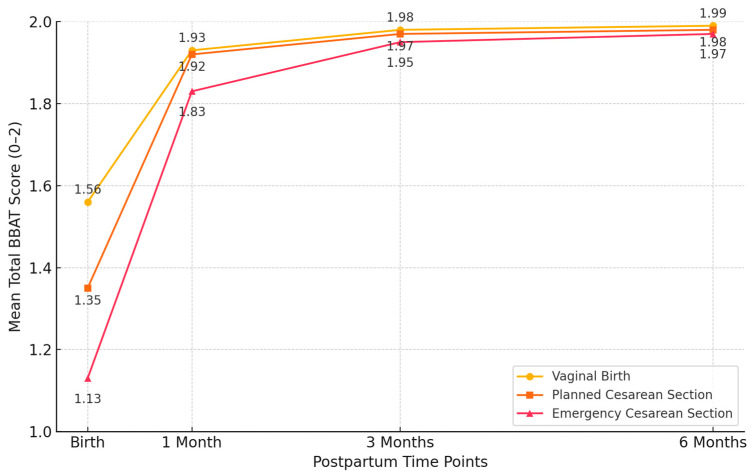
Total Bristol Breastfeeding Assessment Tool (BBAT) scores by delivery method at different time points. BBAT scores range from 0 to 2, with higher scores indicating better breastfeeding technique.

By one month postpartum, these differences diminished, with mean BBAT scores converging across the groups (vaginal: 1.93 ± 0.19; range: 1.5–2.0, planned C/S: 1.92 ± 0.20; range: 1.4–2.0, emergency C/S: 1.83 ± 0.23; range: 1.3–2.0; *p* = 0.068). At three and six months postpartum, BBAT scores remained high and comparable between the groups (*p* = 0.466 and *p* = 0.751, respectively), suggesting that early challenges in breastfeeding technique were overcome with time and support (Figure 3, Table 3).

Repeated measures ANOVA demonstrated significant improvements in BBAT scores within each delivery group over time (*p* < 0.001 for all groups). The emergency cesarean section group exhibited the largest relative improvement in total BBAT scores from birth to six months (74.3% increase), followed by planned C/S (46.7%) and vaginal delivery (27.6%). A significant time × delivery method interaction (*p* < 0.001) indicated differential recovery trajectories among the groups (Table 4).

The correlation analysis revealed significant associations between maternal and perinatal factors and breastfeeding outcomes. Immediate skin-to-skin contact was negatively correlated with NICU admission (r = −0.798, *p* < 0.001), and also showed a weak but statistically significant negative correlation with BBAT scores at birth (r = −0.117, *p* = 0.032), indicating a potential link with early breastfeeding effectiveness. Baseline BBAT scores positively correlated with newborn birth weight (r = 0.377, *p* < 0.001). Interestingly, a positive correlation was also found with NICU admission (r = 0.321, *p* < 0.001), which may reflect increased breastfeeding support efforts or maternal motivation in cases where infants required NICU care. The regression analysis revealed that maternal education level accounted for only 3.2% of the variance in BBAT scores at birth and 2.7% of the variance in breastfeeding continuation at six months postpartum (adjusted R^2^ values). These findings were not statistically significant (*p* > 0.05), indicating limited predictive value. At six months postpartum, these associations had weakened and were no longer statistically significant (Table 5).

## 4. Discussion

This study was conducted to investigate the impact of the method of delivery on breastfeeding initiation rates and breastfeeding duration as well as the rates of immediate skin-to-skin contact and mothers’ education level. Notably, we found the overall breastfeeding initiation rate to be 98.5% in our study group, which is substantially higher than the global average of 41.6% [22]. Although early initiation within one hour postpartum is a key WHO indicator, our findings suggest that structured postpartum support may mitigate initial delays, underscoring the need for tailored lactation programs, particularly for cesarean deliveries. This aligns with previous studies reporting that postpartum support interventions significantly improve breastfeeding outcomes irrespective of delivery method [23]. In parallel, another study reported that the breastfeeding initiation rate in low-resource settings was 81.5%, and the breastfeeding continuation rate at six months postpartum was 70.5% [24].

### 4.1. Mothers’ Demographic Characteristics and Breastfeeding Outcomes

The mothers’ demographic characteristics, such as education level, profession, and pregestational BMI, had an impact on breastfeeding practices. Mothers’ educational level reportedly has a modest secondary effect on breastfeeding outcomes, considering other contributing factors [25], as higher education levels were positively correlated with infant feeding outcomes. Studies conducted in Nigeria and Morocco have reported a positive correlation between higher education levels and increased breastfeeding knowledge among mothers [26,27]. These findings may be attributed to the fact that mothers with higher levels of education potentially become more knowledgeable about breastfeeding and have easier access to breastfeeding support, therefore they tend to have greater awareness about breastfeeding and are more likely to exclusively breastfeed their babies [28]. However, our regression analyses revealed that the mothers’ education level explained only a minimal amount of the variance in breastfeeding behaviors, as detailed in the Results section, suggesting that other factors, such as family support, the accessibility of healthcare services, and maternal motivation, are perhaps more crucial to breastfeeding success.

### 4.2. Breastfeeding Support and Breastfeeding Outcomes

Flexible or supportive practices for breastfeeding in the workplace have been associated with higher rates of breastfeeding among the mothers who work outside the home [27]. The low rates of working mothers (22.5%) and mothers returning to work at three (0.6%) and six (4.4%) months postpartum in our sample may have contributed to the high rates of breastfeeding we observed throughout the study period.

Several studies have shown that the method of delivery significantly affects breastfeeding outcomes, especially during the initial period after birth. One of these studies revealed that those who had an assisted vaginal birth were less likely to breastfeed at three months postpartum compared with those who had an unassisted vaginal birth [29]. Cesarean section deliveries may reportedly delay breastfeeding initiation in infants compared with vaginal deliveries due to difficulties in acquiring the motor skills for effective latching and sucking [30]. Variations in early bonding, skin-to-skin contact, and maternal comfort after cesarean section delivery may also influence breastfeeding outcomes [31]. Basic training specifically designed for mothers with a high BMI may also improve breastfeeding knowledge and skills, enabling women to breastfeed for longer periods [32]. These findings emphasize the critical role of Baby-Friendly Hospital Initiative (BFHI) practices, such as immediate skin-to-skin contact, early rooming-in, and structured lactation counseling, especially for mothers who deliver via cesarean section. Hospitals should ensure that post-cesarean mothers receive targeted support from trained lactation consultants to mitigate early challenges and promote sustained breastfeeding [33,34].

### 4.3. Method of Delivery and Breastfeeding Outcomes

Our findings clearly demonstrated that the method of delivery is a major factor in breastfeeding initiation. Babies born via emergency cesarean section tend to be born with more primitive motor activity at birth than babies born via vaginal delivery and planned cesarean section. Then again, babies’ developmental systems recover well, and early postnatal interventions may also be effective [35]. The differences in breastfeeding rates depending on the method of delivery are most pronounced after birth and gradually become insignificant as motor skills become more consistent at three and six months postpartum, emphasizing the need for targeted postpartum breastfeeding support, particularly for mothers and infants who experienced an emergency cesarean delivery [36].

Our findings regarding the impact of the delivery method on breastfeeding initiation are consistent with the existing literature. Babies born by cesarean section, especially emergency cesarean section, often have difficulty initiating breastfeeding, primarily due to delayed skin-to-skin contact and the effects of anesthesia on neonatal reflexes [37,38]. It is important to acknowledge that the timing of breastfeeding initiation within the first hour postpartum, although widely recommended by WHO and other global health organizations as a key indicator, may not universally predict long-term breastfeeding success. The variability in maternal and neonatal factors should be considered when interpreting this outcome. Nevertheless, early initiation has been associated with improved neonatal survival and reduced morbidity, making it a critical component of postpartum care [6]. In our cohort, while early initiation rates varied significantly by delivery method, the convergence of breastfeeding continuation rates at later time points suggests that initial delays can be mitigated through structured postpartum support. On the other hand, our study differs from previous studies, which reported that the adverse effects of cesarean section on the breastfeeding outcomes of infants were permanent, as we showed that the adverse impact of cesarean section on the breastfeeding outcomes of infants became insignificant at three and six months postpartum compared with vaginal delivery. As a matter of fact, postnatal support and external factors reportedly mediate long-term breastfeeding success [39]. Furthermore, our data indicate that although cesarean delivery initially delays breastfeeding initiation, by six months postpartum, breastfeeding continuation rates were comparable across all delivery methods, highlighting that the delivery mode’s impact diminishes over time. Moreover, our findings reporting a relatively weak predictive value for mothers’ education level on breastfeeding outcomes also suggests that breastfeeding success depends on many factors. Finally, our effective use of BBAT in a clinical setting to determine how the method of delivery and maternal factors affect breastfeeding outcomes shows that breastfeeding assessment tools can be effectively integrated into routine postpartum care.

Our study’s primary strength lies in its prospective cohort design, which minimizes recall bias and allows for the structured, time-sensitive observation of breastfeeding behaviors during the critical postpartum period. The systematic follow-up of participants from birth to six months allowed for the capture of temporal changes in breastfeeding dynamics and provided a more robust understanding of early feeding practices compared with cross-sectional studies.

Using validated BBAT as an assessment tool further strengthened our methodological rigor. Such tools ensure the standardized evaluation of key breastfeeding components, enhancing the reliability and reproducibility of the findings. Additionally, by comparatively reviewing various delivery methods, i.e., vaginal delivery, planned cesarean section, and emergency cesarean section, we address a significant gap in the existing literature and provide a comprehensive perspective on how different obstetric contexts affect breastfeeding outcomes.

In addition to its strengths, this study has several limitations. Firstly, the predictor variables were intentionally kept narrow to focus on delivery method and immediate postpartum breastfeeding outcomes. Secondly, the data on breastfeeding practices beyond BBAT scoring were self-reported, introducing potential recall and social desirability biases. Thirdly, the single-center design limits the generalizability to other populations and institutional settings. Finally, as an observational study, randomization was not feasible, and the delivery method was dictated by clinical indications, limiting the ability to infer causality. These limitations reflect real-world clinical practice and highlight the need for future multicenter randomized trials with objective measures of breastfeeding success.

### 4.4. Limitations

Despite its strengths, this study has several limitations. As it was conducted at a single tertiary-level Baby-Friendly hospital, the generalizability of the findings to other clinical settings or healthcare systems may be limited. While the prospective design and structured follow-up improved data reliability, residual confounding factors such as maternal motivation, psychological well-being, or informal family support may still have influenced breastfeeding outcomes. Additionally, self-reported breastfeeding practices are prone to recall and social desirability biases, particularly over long-term follow-up. The Bristol Breastfeeding Assessment Tool (BBAT), although validated, primarily assesses the technical aspects of feeding and may not capture broader psychosocial dimensions. Moreover, the early initiation of breastfeeding within the first hour postpartum, while a key WHO indicator, may not universally predict long-term success, which is a contextual limitation. Finally, as an observational study, randomization was not feasible, and the delivery method was dictated by clinical indications, limiting causal inference. Recognizing these limitations is crucial for interpreting our results and guiding future multicenter randomized trials with diverse populations and objective measures of breastfeeding success.

## 5. Conclusions

This study highlights that while cesarean delivery—particularly emergency cesarean section—can initially delay breastfeeding initiation, these early challenges do not persist beyond the postpartum period. By six months, breastfeeding continuation rates were comparable across all delivery methods, underscoring the critical role of structured postpartum support. These findings reinforce the importance of Baby-Friendly Hospital Initiative (BFHI) practices, including immediate skin-to-skin contact, effective pain management, and lactation counseling, in ensuring equitable breastfeeding outcomes. Future efforts should focus on developing targeted post-cesarean support programs and evaluating their long-term impact through multicenter studies.

## Figures and Tables

**Table 1:** Sociodemographic characteristics of the study population (n = 338).

Characteristics	Vaginal Birth (n = 83)	Planned C/S (n = 178)	Emergency C/S (n = 77)
Maternal Age (years)	28.4 ± 4.1 (16–42)	28.3 ± 4.3 (17–51)	27.9 ± 4.2 (16–43)
Pregestational BMI (kg/m^2^)	25.9 ± 3.1 (19.1–34.2)	26.4 ± 3.2 (19.8–34.8)	26.1 ± 3.3 (19.6–34.5)
Weight Gain During Pregnancy (kg)	14.6 ± 4.5 (7–26)	15.0 ± 4.7 (8–27)	14.7 ± 5.0 (7–25)
Education Level: Primary	39 (47.0%)	76 (42.7%)	34 (44.2%)
Education Level: Secondary	16 (19.3%)	34 (19.1%)	13 (16.9%)
Education Level: High School	16 (19.3%)	38 (21.3%)	12 (15.6%)
Education Level: University	12 (14.4%)	30 (16.9%)	18 (23.3%)
Mother’s Profession: Healthcare Worker	4 (4.8%)	11 (6.2%)	7 (9.1%)
Mother’s Profession: Housewife	65 (78.3%)	136 (76.4%)	61 (79.2%)
Mother’s Profession: Civil Servant	3 (3.6%)	10 (5.6%)	2 (2.6%)
Mother’s Profession: Teacher	5 (6.0%)	11 (6.2%)	4 (5.2%)
Mother’s Profession: Factory Worker	6 (7.2%)	10 (5.6%)	3 (3.9%)

Note: BMI = Body Mass Index. Data presented as frequency (percentage) for categorical variables and mean ± standard deviation (SD) for continuous variables.

**Table 2:** Breastfeeding outcomes by delivery method (vaginal birth, planned cesarean section, and emergency cesarean section).

Breastfeeding Outcomes	Vaginal Birth (n = 83)	Planned C/S (n = 178)	Emergency C/S (n = 77)	*p*
Initiation of Breastfeeding within 1 h	85.5% (71/83)	57.9% (103/178)	64.9% (50/77)	**<0.001**
Breastfeeding Continuation at 1 Month	98.8% (82/83)	98.3% (175/178)	98.7% (76/77)	0.945
Breastfeeding Continuation at 3 Months	96.4% (80/83)	96.1% (171/178)	94.8% (73/77)	0.853
Breastfeeding Continuation at 6 Months	95.2% (79/83)	94.4% (168/178)	94.8% (73/77)	0.880
Formula Supplementation at 1 Month	13.3% (11/83)	16.9% (30/178)	16.9% (13/77)	0.724
Formula Supplementation at 6 Months	16.9% (14/83)	18.0% (32/178)	18.2% (14/77)	0.967
Complementary Feeding at 6 Months	24.1% (20/83)	24.2% (43/178)	24.7% (19/77)	0.995

Note: Data presented as n (%). Bold *p*-values indicate statistical significance (*p* ≤ 0.05). Chi-square tests were used for comparing proportions between groups. World Health Organization (WHO) [6] defines early initiation of breastfeeding as breastfeeding within the first hour after birth.

**Table 3:** Bristol Breastfeeding Assessment Tool (BBAT) scores by delivery method at different time points.

Subscale	Time Point	Vaginal Birth (n = 83)	Planned C/S (n = 178)	Emergency C/S (n = 77)	*p*
Positioning	Birth	1.60 ± 0.28 (1.0–2.0)	1.42 ± 0.30 (0.9–1.9)	1.19 ± 0.35 (0.7–1.8)	**<0.001**
Positioning	1 Month Postpartum	1.95 ± 0.15 (1.6–2.0)	1.93 ± 0.18 (1.5–2.0)	1.84 ± 0.21 (1.4–2.0)	**0.045**
Positioning	3 Months Postpartum	1.99 ± 0.05 (1.8–2.0)	1.98 ± 0.07 (1.7–2.0)	1.96 ± 0.10 (1.6–2.0)	0.321
Positioning	6 Months Postpartum	2.00 ± 0.00 (2.0–2.0)	1.99 ± 0.05 (1.9–2.0)	1.98 ± 0.08 (1.7–2.0)	0.658
Attachment	Birth	1.52 ± 0.33 (0.9–2.0)	1.33 ± 0.35 (0.8–1.9)	1.08 ± 0.39 (0.6–1.7)	**<0.001**
Attachment	1 Month Postpartum	1.92 ± 0.17 (1.5–2.0)	1.91 ± 0.19 (1.4–2.0)	1.82 ± 0.24 (1.3–2.0)	**0.039**
Attachment	3 Months Postpartum	1.98 ± 0.07 (1.8–2.0)	1.97 ± 0.09 (1.6–2.0)	1.94 ± 0.11 (1.6–2.0)	0.284
Attachment	6 Months Postpartum	1.99 ± 0.06 (1.8–2.0)	1.98 ± 0.08 (1.7–2.0)	1.96 ± 0.10 (1.7–2.0)	0.710
Sucking	Birth	1.59 ± 0.29 (1.0–2.0)	1.39 ± 0.31 (0.8–1.9)	1.16 ± 0.38 (0.7–1.8)	**<0.001**
Sucking	1 Month Postpartum	1.94 ± 0.16 (1.6–2.0)	1.92 ± 0.20 (1.5–2.0)	1.83 ± 0.23 (1.4–2.0)	**0.042**
Sucking	3 Months Postpartum	1.99 ± 0.04 (1.8–2.0)	1.98 ± 0.06 (1.7–2.0)	1.96 ± 0.09 (1.7–2.0)	0.299
Sucking	6 Months Postpartum	2.00 ± 0.00 (2.0–2.0)	1.99 ± 0.04 (1.9–2.0)	1.98 ± 0.07 (1.8–2.0)	0.688
Swallowing	Birth	1.54 ± 0.35 (0.9–2.0)	1.29 ± 0.36 (0.7–1.8)	1.09 ± 0.37 (0.7–1.7)	**<0.001**
Swallowing	1 Month Postpartum	1.91 ± 0.18 (1.5–2.0)	1.90 ± 0.21 (1.3–2.0)	1.81 ± 0.25 (1.3–2.0)	**0.037**
Swallowing	3 Months Postpartum	1.98 ± 0.08 (1.8–2.0)	1.97 ± 0.10 (1.6–2.0)	1.94 ± 0.12 (1.6–2.0)	0.317
Swallowing	6 Months Postpartum	1.99 ± 0.06 (1.8–2.0)	1.98 ± 0.08 (1.7–2.0)	1.96 ± 0.09 (1.7–2.0)	0.703

Note: Data presented as mean ± standard deviation. Bristol Breastfeeding Assessment Tool (BBAT) scores range from 0 to 2, with higher scores indicating better breastfeeding technique. Bold *p*-values indicate statistical significance (*p* ≤ 0.05). ANOVA was used for comparing means between groups.

**Table 4:** Within-group temporal changes in BBAT components by delivery method: repeated measures ANOVA results.

Delivery Method	BBAT Component	F-Value	df	*p*-Value	η^2^	Absolute Change (Birth→6th Month)	Relative Change
Vaginal Birth (n = 83)	Positioning	64.7	−3246	**<0.001**	0.441	+0.41	+25.9%
	Attachment	47.1	−3246	**<0.001**	0.365	+0.36	+22.1%
	Sucking	84.8	−3246	**<0.001**	0.508	+0.47	+30.9%
	Swallowing	91.5	−3246	**<0.001**	0.527	+0.49	+32.9%
	**Total BBAT Score**	**68.5**	**−3246**	**<0.001**	**0.455**	**+0.43**	**+27.6%**
Planned Cesarean (n = 178)	Positioning	249.3	−3531	**<0.001**	0.584	+0.55	+38.5%
	Attachment	296.0	−3531	**<0.001**	0.626	+0.60	+43.5%
	Sucking	386.2	−3531	**<0.001**	0.686	+0.68	+51.9%
	Swallowing	411.6	−3531	**<0.001**	0.699	+0.71	+55.9%
	**Total BBAT Score**	**330.0**	**−3531**	**<0.001**	**0.651**	**+0.63**	**+46.7%**
Emergency Cesarean (n = 77)	Positioning	182.0	−3228	**<0.001**	0.705	+0.73	+58.9%
	Attachment	262.7	−3228	**<0.001**	0.776	+0.87	+79.1%
	Sucking	247.9	−3228	**<0.001**	0.765	+0.84	+73.7%
	Swallowing	297.9	−3228	**<0.001**	0.797	+0.92	+87.6%
	**Total BBAT Score**	**244.1**	**−3228**	**<0.001**	**0.762**	**+0.84**	**+74.3%**

Note: Bold *p*-values indicate statistical significance (*p* ≤ 0.05). Repeated measures ANOVA demonstrated significant within-group temporal improvements in all BBAT components across delivery methods. Mixed ANOVA revealed three critical effects: time (F = 10423.35, *p* < 0.001) confirming significant temporal improvement across all groups; delivery method (F = 29.81, *p* < 0.001) establishing between-group differences; and time × delivery method interaction (F = 12.60, *p* < 0.001) demonstrating differential recovery patterns by delivery method. The emergency cesarean section group exhibited the most pronounced recovery trajectory (74.3% total improvement), followed by the planned cesarean section (46.7%) and vaginal delivery (27.6%) groups. The significant interaction effect confirms that while all delivery methods achieved substantial improvements in BBAT scores from birth to six months, the rate and magnitude of recovery varied significantly by delivery type, with convergence achieved by six months postpartum. These findings demonstrate that initial breastfeeding challenges associated with cesarean delivery are transient and completely recoverable with appropriate support. BBAT = Bristol Breastfeeding Assessment Tool; df = degrees of freedom; η^2^ = partial eta squared (effect size). ↑ indicates improvement from birth to 6 months postpartum; η^2^ = partial eta squared; *p*-values indicate statistical significance of changes over time.

**Table 5:** Correlation analysis of key factors affecting breastfeeding outcomes.

Variables	Immediate Skin-to-Skin Contact	Newborn Birth Weight	NICU Admission	BBAT Score at Birth
Pregestational BMI	−0.113 (0.056)	**0.164 (0.003)** *	−0.097 (0.075)	−0.086 (0.115)
Weight Gain During Pregnancy	−0.103 (0.059)	**0.274 (<0.001)** **	−0.058 (0.287)	−0.073 (0.180)
Vaginal Birth Duration	**−0.296 (0.007)** **	**0.287 (0.009)** **	−0.174 (0.116)	0.182 (0.100)
Immediate Skin-to-Skin Contact	1	**−0.411 (<0.001)** **	**−0.798 (<0.001)** **	**−0.117 (0.032)** *
Newborn Birth Weight	**−0.411 (<0.001)** **	1	**0.346 (<0.001)** **	**0.377 (<0.001)** **
NICU Admission	**−0.798 (<0.001)** **	**0.346 (<0.001)** **	1	**0.321 (<0.001)** **
BBAT Score at 1 Month	−0.089 (0.103)	0.377 (<0.001) **	0.321 (<0.001) **	0.413 (<0.001) **
BBAT Score at 3 Months	−0.278 (<0.001) **	0.281 (0.010) **	0.185 (0.001) **	0.317 (<0.001) **
BBAT Score at 6 Months	−0.076 (0.162)	0.062 (0.255)	0.038 (0.491)	0.073 (0.181)

Note: Data presented as correlation coefficient r (*p*-value). * indicates *p* < 0.05, ** indicates *p* < 0.01. BBAT = Bristol Breastfeeding Assessment Tool; BMI = Body Mass Index; NICU = Neonatal Intensive Care Unit. Negative correlation with NICU admission indicates fewer admissions (as 1 = yes, 2 = no in the original coding). Bold values indicate statistically significant correlations. Correlation coefficients (r) are Pearson’s correlation values. *p*-values indicate statistical significance.

## Data Availability

The data presented in this study are available on request from the corresponding author due to national restrictions.

## Abbreviations

The following abbreviations are used in this manuscript:

BBAT	Bristol Breastfeeding Assessment Tool
BMI	Body Mass Index
BFHI	Baby-Friendly Hospital Initative
NICU	Neonatal Intensive Care Unit
WHO	World Health Organization
